# Berberine Inhibits Intestinal Polyps Growth in Apc (min/+) Mice via Regulation of Macrophage Polarization

**DOI:** 10.1155/2016/5137505

**Published:** 2016-07-17

**Authors:** Meiyu Piao, Hailong Cao, NaNa He, Boli Yang, Wenxiao Dong, Mengque Xu, Fang Yan, Bing Zhou, Bangmao Wang

**Affiliations:** ^1^Department of Gastroenterology, General Hospital, Tianjin Medical University, Tianjin, China; ^2^Department of Gastroenterology, The Four Zero One Hospital of the People's Liberation Army, Qingdao, China; ^3^Department of Digestive Diseases, General Hospital, Jincheng Coal Group, Jincheng, Shanxi, China; ^4^Division of Gastroenterology, Hepatology and Nutrition, Department of Pediatrics, Vanderbilt University Medical Center, Nashville, TN 37232, USA; ^5^Department of Gastroenterology, Tanggu Traditional Chinese Medicine Hospital of Tianjin Binhai New Area, No. 90 Hangzhou Road, Tanggu, Binhai New Area, Tianjin 300450, China

## Abstract

Antitumor effect of berberine has been reported in a wide spectrum of cancer, however, the mechanisms of which are not fully understood. The aim of this study was to investigate the hypothesis that berberine suppresses tumorigenesis in the familial adenomatous polyposis (FAP) by regulating the macrophage polarization in Apc (min/+) mouse model. Berberine was given to Apc (min/+) mice for 12 weeks. Primary macrophages were isolated; after berberine treatment, the change in signaling cascade was determined. The total number and size of polyps were reduced remarkably in berberine group, compared with control group. A significant decrease in protein levels of F4/80, mannose receptor (MR), and COX-2 in stroma of intestinal polyps and an increase in the level of iNOS were observed after berberine treatment. The mRNA level of MR and Arg-1 in berberine group was significantly lower than those in IL-10 or IL-4 group, while no significant difference in mRNA levels of iNOS and CXCL10 was observed. The migration and invasiveness assays* in vitro* showed that berberine could reduce the capability of migration and invasiveness. These findings suggest that berberine attenuates intestinal tumorigenesis by inhibiting the migration and invasion of colorectal tumor cells via regulation of macrophage polarization.

## 1. Introduction

Familial adenomatous polyposis (FAP), which is diagnosed by detection of adenomatous polyps, is a hereditary tumorous predisposition syndrome; it is caused by germline mutation, especially in the adenomatous polyposis coli (APC) genes [1]. In patients with FAP, huge amount of polyps appears in the colorectal area during second and third decades. It is reported that APC gene mutation leads to FAP and further results in the development of multiple colorectal adenomas at an early age, which finally causes CRC [2, 3].

Berberine is an alkaloid isolated from* Coptis chinensis*, which is commonly used in Chinese traditional medicine to treat gastrointestinal disorders [4]. Recently, berberine is demonstrated to have multiple pharmacological activities, including anticancer effect [5–7]. Our previous work demonstrated that berberine could inhibit the proliferation of colorectal cancer cells by inhibiting wnt/*β*-catenin activity, where nuclear translocation of *β*-catenin was disrupted [8]. Additionally, anti-inflammatory effect of berberine was documented in several disease models, such as diabetes [9, 10]. However, the anti-inflammatory effect of berberine on intestinal tumor is poorly understood.

In this study, we investigated the effect of berberine on macrophage polarization in the development of FAP. The Apc (min/+) mice model, a well established spontaneous intestinal tumorigenesis mouse model, was applied. The findings suggested that berberine suppresses tumorigenesis in the familial adenomatous polyposis (FAP) by regulating the macrophage polarization.

## 2. Materials and Methods

### 2.1. Establishment of Animal Model

Five four-week-old Apc (min/+) mice and five age-matched C57BL/6 mice were purchased from Model Animal Research Center of Nanjing University. As described previously [11], berberine was given by drinking water (0.1% w/v) daily for up to 12 weeks. All animals were maintained on a 12-hour light/dark cycle and were fed* ad libitum*. The small intestine and colon were taken from mice under anesthesia for further study. The present study was performed with the approval of the Committee for Animal Research at Tianjin Medical University.

### 2.2. Cell Culture

Mouse peritoneal macrophages (MPMΦ) were isolated from wild-type C57BL/6 mice as described previously [12], with minor modifications. MPMΦ, mouse macrophage-like cells RAW264.7, and HT-29 cells purchased from ATCC were plated and cultured in DMEM with 10% FBS at 37°C with 5% CO_2_. In some experiments, MPMΦ were treated with 20 ng/mL IL-4 or IL-10 with/without berberine or 20 ng/mL INF-*γ*. RAW264.7 cells were replated on a 0.4 *μ*m insert for noncontacting coculture with HT-29.

### 2.3. Real-Time PCR Analysis

Expression levels of mRNAs were evaluated by real-time PCR as previously described [8], with slight modification. Total RNA was isolated with TRIzol reagent (Invitrogen, USA). Real-time PCR was performed on a GeneAmp 7300 Real-Time PCR System (Applied Biosystems, Foster City, CA, USA) with SYBR® Premix Ex Taq*™* kit (TaKaRa). The primers sequences were listed in Table 1.

### 2.4. Western Blot Analysis

Western blotting was performed on SDS-PAGE Electrophoresis System as described previously [12]. Briefly, 30 *μ*g protein of each sample was resuspended in a reduced sample buffer and then was electrophoresed on a 10% Tris gel with running buffer; then it was blotted to PVDF membrane. Membranes were blocked with 5% nonfat milk and incubated overnight with an anti-COX-2 antibody (Abcam) or with anti-*β*-actin antibody (Sigma). Immunoreactive bands were detected with ECL-Plus (GE Healthcare) following incubation with secondary antibodies. The band intensity was quantified densitometrically using ImageJ software.

### 2.5. Immunohistochemistry

Tissues isolated from mice under anesthesia were fixed with 4% PFA overnight; they were washed three times with PBS. After being embedded in paraffin, tissues were sectioned into 5 *μ*m slices and were stained with anti-COX-2, anti-iNOS, anti-MR, and anti-F4/80 antibodies overnight at 4°C. The following steps were then performed with the immunostaining kit according to the manufacturer's instructions.

### 2.6. Cell Proliferation

MTT (Sigma, USA) were purchased commercially. The cell proliferation was determined according to manufacturer's instruction.

### 2.7. Cell Migration Assay

The migration of HT-29 cells was detected using 24-well Transwell inserts (Corning, USA) coated with 30 *μ*g of Matrigel (BD Biosciences), as described previously [13]. Briefly, berberine-pretreated RAW264.7 cells were seeded into 24-well culture plates at a density of 1 × 10^5^ cells per well. In addition, 1 × 10^4^ HT-29 cells were seeded in the upper chamber, the upper surface of which was coated with a thin layer of Matrigel (5 mg/mL). After incubation for 24 h, the membranes were removed and were stained with hematoxylin and eosin (H&E), and the numbers of cells that invaded into Matrigel were counted in three randomly selected fields under light microscopy.

Scratch wound assays were applied to HT-29 cells as described [14]. Briefly, HT-29 cells were seeded at a density of 5 × 10^5^ cells into 6-well-plate. A linear mechanical scratch wound was generated using a 20 *μ*L plastic pipette tip, after noncontacting coculture with berberine-pretreated RAW264.7. The cells were exposed to 20 *μ*M or 40 *μ*M berberine for 12 h or 24 h. Phase contrast images were taken at 0 h, 12 h, and 24 h after scratching.

### 2.8. Statistical Analyses

The* in vitro* tests were performed three times independently in triplicate. Data were expressed as the means ± SEM and were subjected to one-way ANOVA. For multiple datasets, a post hoc multiple comparison (two-tailed multiple *t*-test with Bonferroni correction) was applied.

## 3. Results

### 3.1. Berberine Treatment Leads to Alteration in the Proportion and Distribution of the Macrophages in the Intestinal Polyps

**Figure 1:**
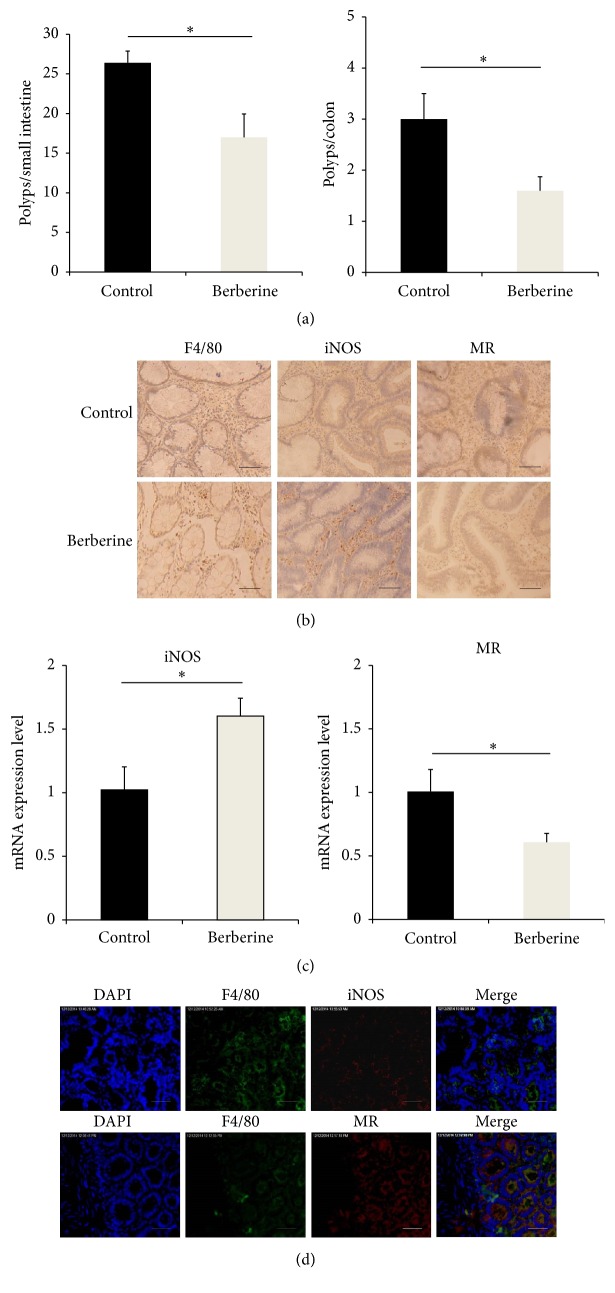
Berberine treatment leads to alteration in the proportion and distribution of the macrophages in the intestinal polyps. (a) Tumor numbers were counted in the small intestine and colon of Apc (min/+) mice, ^*∗*^ *p* < 0.05. (b) Immunohistochemistry staining of F4/80, iNOS, and MR in the small intestine was performed. Five images were randomly selected and the cell numbers were calculated. Bar = 100 *μ*m. (c) Total mRNA was extracted and mRNA expression level was measured by real-time PCR, ^*∗*^ *p* < 0.05. (d) Fluorescent immunocytochemistry was performed to assess protein expression of iNOS, MR, and F4/80. Bar = 200 *μ*m. *N* = 5.

Macrophages play an important role in invasion, proliferation, and metastasis of tumor cells. Previously, we reported that berberine could attenuate intestinal tumor development in Apc (min/+) [12] (Figure 1(a)). Then, the effect of berberine on macrophages number in the intestinal villi was identified by F4/80, iNOS, and MR immunohistochemistry in the small intestine. Compared with control group, the number of F4/80+ and MR+ macrophages was significantly decreased in the small intestine of berberine group, while the number of iNOS+ macrophages was increased significantly (Figure 1(b)). Accordingly, an elevation in mRNA level of iNOS together with a decrease in MR level was observed in berberine group, compared with control group (Figure 1(c)). Simultaneously, immunohistochemical double-staining results showed that the majority of the iNOS and MR expressions were colocalized in F4/80+ cells (Figure 1(d)).

### 3.2. Berberine Reduces Inflammation Level in the Intestinal Polyps

**Figure 2:**
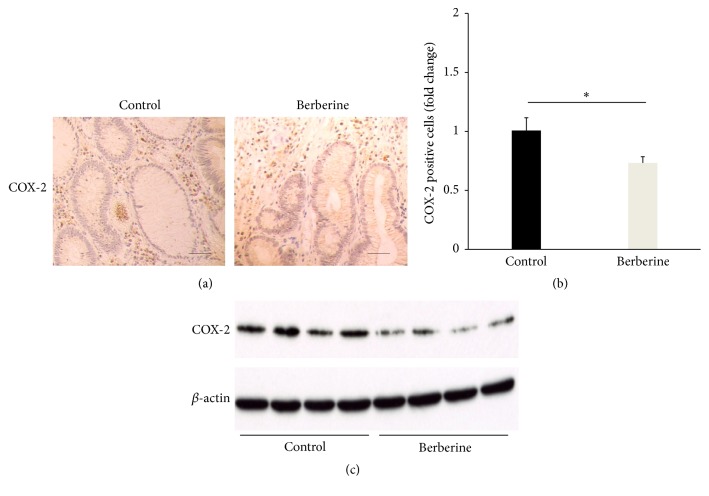
Berberine reduces inflammation level in the intestinal polyps. (a, b) Immunohistochemistry staining of COX-2 in the small intestine. Bar = 50 *μ*m. (c) Representative immunoblots and quantitative densitometric analysis of COX-2 in the small intestine. *N* = 3, ^*∗*^ *p* < 0.05.

The anti-inflammatory effect of berberine on the development of intestinal polyps was investigated afterward by determining the protein level of COX-2. Immunohistochemistry result showed that significant reduction of COX-2 positive cells was observed after berberine treatment (Figures 2(a) and 2(b)), whereas a decrease in COX-2 protein level was confirmed in berberine group as well (Figure 2(c)).

### 3.3. Berberine Induces M2 to M1 Phenotype Switching

**Figure 3:**
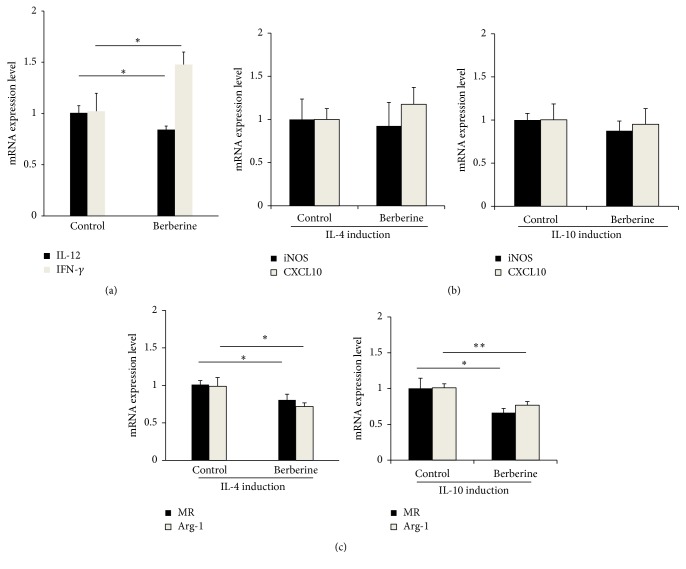
Berberine induces M2 to M1 phenotype switching. The mRNA expression level was measured by real-time PCR. The expression of (a) IL-12 and IFN-*γ*, (b) iNOS and CXCL10, and (c) MR and Arg-1 in primary isolated MPMΦ. *N* = 3, ^*∗*^ *p* < 0.05, and ^*∗∗*^ *p* < 0.001.

To verify whether berberine affects macrophage polarization, real-time PCR was performed to detect mRNA level of IL-12, a marker of M2 macrophage, and IFN-*γ*, a marker of M1 macrophage. After berberine treatment, IL-12 mRNA level was decreased, while IFN-*γ* was elevated significantly in the intestinal polyps (Figure 3(a)).

To further confirm the effect of berberine on macrophage polarization, we isolated MPMΦ and then induced these cells into M2 macrophage by IL-4 or IL-10. No change in iNOS and CXCL10 mRNA level was observed. In contrast, mRNA levels of MR and Arg-1 were significantly suppressed, which was induced by IL-4 or IL-10 (Figures 3(b) and 3(c)).

### 3.4. Berberine Suppresses Invasion and Migration of Tumor Cells via Alteration in Macrophage Polarization

**Figure 4:**
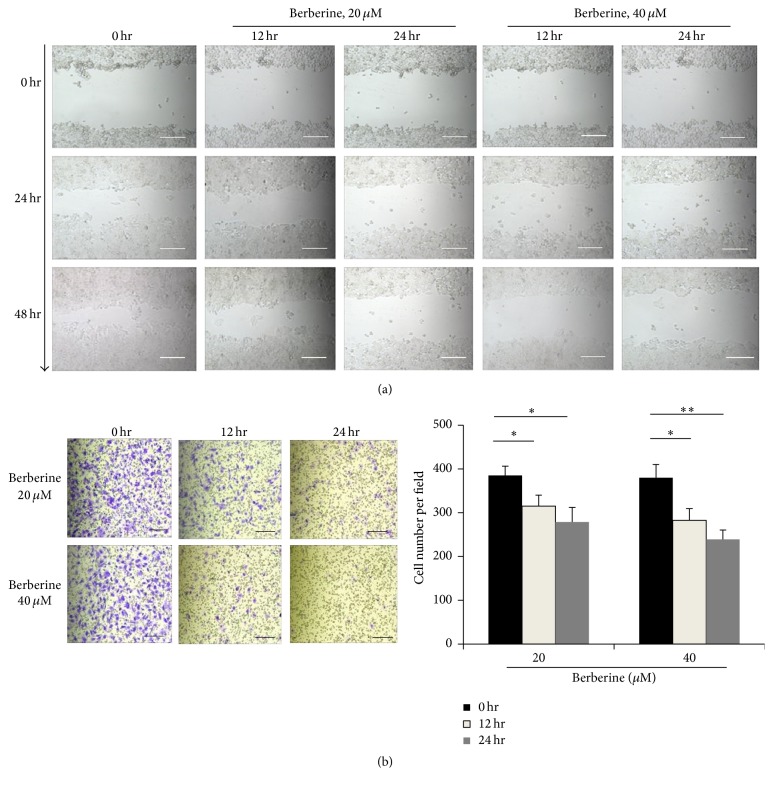
Berberine suppresses invasion and migration of tumor cells via alteration in macrophage polarization. (a) Migration of berberine-induced M2 macrophage by* in vitro* scratch assay, bar = 100 *μ*m. (b) Migration of tumor cells was assessed in Transwell cell culture chambers, bar = 100 *μ*m. *N* = 5, ^*∗*^ *p* < 0.05, and ^*∗∗*^ *p* < 0.001.

To investigate whether berberine affects invasion and migration of tumor cells, noncontacting coculture of HT-29 cells and RAW264.7 was conducted, in which M2 polarization was induced by berberine. Firstly, we performed scratch assay to assess the effect of berberine-induced M2 macrophage on HT-29 cells migration. HT-29 cells migration, which was confirmed after being cocultured with IL-4 induced M2 macrophage, was suppressed when M2 macrophage was treated with berberine (Figure 4(a)). Then, Transwell migration assay was performed to verify the effect of berberine on tumor cell migration. A remarkable decrease of cell migration was demonstrated in berberine treated M2 macrophage group in a dose-dependent manner (Figure 4(b)).

### 3.5. Berberine Treatment Could Reduce NADPH Oxidase (NOX) mRNA Level in Macrophages

**Figure 5:**
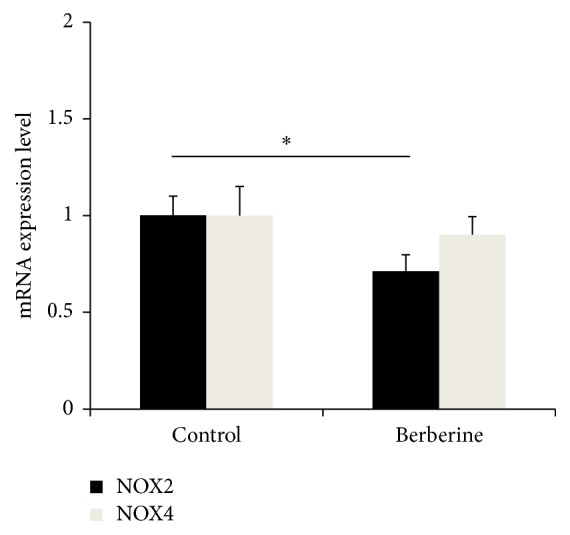
Berberine treatment could reduce NADPH Oxidase (NOX) mRNA level in macrophage. The expression levels of NOX2 and NOX4 were significantly decreased compared to control group. *N* = 3, ^*∗*^ *p* < 0.05.

Since reactive oxygen species (ROS) could specifically affect the polarization of M2 macrophage, we measured mRNA expression levels of NOX2 and NOX4 in the M2 macrophage. After berberine treatment, mRNA level of NOX2 was significantly reduced, whereas no change in mRNA level of NOX4 was observed (Figure 5).

## 4. Discussion

Chinese herbal medicine is an important part of alternative medicine. Berberine is a bioactive component originated from* Coptidis Rhizome*; it has been widely used as immunomodulatory agent for treating autoimmune disease including inflammatory bowel disease and ulcerative colitis, as well as colorectal cancer [15–17]. In this study, antitumor effect of berberine was demonstrated in Apc (min/+) mice model, which was in accordance with our previous study. In the intestinal polyps of Apc (min/+), macrophage infiltration was reduced after berberine treatment, accompanied by a decreased M2 polarization.* In vitro* study demonstrated that invasion and migration of tumor cells were significantly decreased when they were cocultured with berberine treated macrophages. Interestingly, expression of NOX2 in M2 macrophage was suppressed by berberine, whereas expression of NOX4 was not affected. These findings indicated that berberine exhibited antitumor effects possibly by reducing expression of NOX2, which further affected polarization of macrophages.

Macrophages play an important role in invasion into normal tissues, proliferation and survival, and metastasis of tumor cells [18]. Previously, COX-2 inhibition was reported to cause the loss of the M2 macrophages phenotype, which may assist prevention of cancer metastasis [19]. Our results showed that a switch from M2 to M1 phenotype was induced by berberine, and COX-2 expression was decreased, accordingly. These data suggested that berberine may cause macrophages polarization via regulating the COX-2 pathway during the inflammation.

NADPH Oxidase, a major source of reactive oxygen species (ROS) produced in response to stimuli, was an important factor involved in M1 macrophage differentiation [20–22]. It was reported that NOX2 and/or NOX4 derived ROS would be suppressed by berberine in the macrophages [23, 24], where anti-inflammatory effect of berberine was demonstrated. In this study, we observed that mRNA level of NOX2, but not NOX4, was reduced by berberine, in accordance with macrophage differentiation. In addition, COX-2 expression was reported to be regulated by NADPH Oxidase/ROS pathway in different cell types [25, 26], which indicated that M2 to M1 phenotype switch induced by berberine would be regulated through the mechanism of NADPH Oxidase/ROS/COX-2 signaling pathway. Further investigation is needed to clarify how berberine regulates the expression level of NADPH Oxidase.

In conclusion, berberine suppressed intestinal tumorigenesis in the Apc (min/+) mice model. This antitumor effect of berberine is possibly mediated by a reduction in M2 macrophage polarization via NADPH Oxidase/ROS/COX-2 signaling pathway.

## Figures and Tables

**Table 1:** The sense and antisense primers.

	Sense	Antisense
MR	GGTGCTACTCCGAACAACAG	ACCGTGGCTGAAAGTTCCT
iNOS	TGAACCCCAAGAGTTTGACC	TGCTGAAACATTTCCTGTGC
IFN-*γ*	TACTGCCACGGCACAGTCATTGAA	GCAGCGACTCCTTTTCCGCTTCCT
IL-10	GGTTGCCAAGCCTTATCGGA	ACCTGCTCCACTGCCTTGCT
CXCL10	TACTGCCACGGCACAGTCATTGAA	GCAGCGACTCCTTTTCCGCTTCCT
Arg-1	GGTTGCCAAGCCTTATCGGA	ACCTGCTCCACTGCCTTGCT
GAPDH	TGTGTCCGTCGTGGATCTGA	CCTGCTTCACCACCTTCTTGA
*β*-actin	GTCCCTCACCCTCCCAAAAG	GCTGCCTCAACACCTCAACCC
